# Notes on the Use of Bacteriological Methods of Diagnosis in Private Practice.—II

**Published:** 1898-09-24

**Authors:** J. O. Symes

**Affiliations:** Bacteriologist to the Royal Infirmary, Bristol


### Medical Progress and Hospital Clinics.

[*The Editor will be glad to receive offers of co-operation and contributions from members of the profession. All letters should be addressed to* The Editor, at the Office, 28 & 29, Southampton Street, Strand, London, W.C.]

### NOTES ON THE USE OF BACTERIOLOGICAL METHODS OF DIAGNOSIS IN PRIVATE PRACTICE.—II.

By J. O. Symes, M.D.Lond., Bacteriologist to the Royal Infirmary, Bristol.

**Diseases of the Serous Membranes.**—Effusions into serous cavities are frequently caused by the presence of micro-organisms or their products. Thus a synovitis may be the result of the action of staphylo-, strepto-, or gono-coccus, or a pleurisy or peritonitis be due to infection by the same organisms, or by pneumococcus, or tubercle.

It is obvious that a knowledge of the determining cause will throw much light on both diagnosis, prognosis, and treatment. The fluid for bacteriological examination is best obtained by means of a hypodermic syringe or exploring needle. This is rendered aseptic by boiling for about half an hour, and the skin over the spot to be punctured having been cleansed with turpentine, perchloride of mercury, and spirit, the fluid is then drawn off. The material thus obtained may be utilised in three ways. A series of drops are spread between cover-glasses and stained with methylene blue for cocci, and with Ziehl-Neelsen's stain for tubercle bacilli. If no cocci can be detected by microscopic examination, then the effusion (especially if it be purulent, as in empyema) should be stained for tubercle bacilli, and failure to find either of these organisms may be taken to mean that there are very few present, and that to detect them it is necessary that the fluid should first be centrifugalised. For this purpose the contents of the syringe should be transferred to a sterile test tube or bottle, and dispatched to the laboratory. In several cases we have, by means of the centrifugal machine, been able to detect micro-organisms in fluids which had been previously thought to be sterile. If culture tubes are at hand a drop of pus from the syringe may be received on the needle, and the surface of the medium inoculated. Pneumococci and tubercle bacilli, however, do not grow easily on ordinary media, and if the method of cultures only be relied upon these organisms might escape detection. It is, therefore, always necessary to check results by having cover-glass preparations.

In connection with empyemata, it is worthy of notice that in tuberculous affections of the pleura it is more common for the fluid to be serous, and in this, after centrifugalising, tubercle bacilli are often found in large numbers. Pneumococcal empyemata may be cured by simple aspiration; but an empyema, due to staphylo- or strepto-cocci, will almost certainly require to be drained.

**General Infections—Examination of Blood.**—There are a large number of diseases in which the specific micro-organism is free in the general circulation. These organisms may be, however, so few in number as to give little hope of successfully detecting them in the small quantity of blood that can be obtained. Amongst the disorders in which this method is most successfully employed are pyæmia and septicæmia, diarrhœa, ulcerative endocarditis, malaria, and, to a lesser extent, enteric fever and anthrax.

Streptococci are sometimes to be detected in the blood in cases of septicæmia, ulcerative endocarditis, puerperal fever, and zymotic diarrhœa. Their presence would point to the advisability of treatment by anti-streptococcic serum. No anti-staphylococcic serum is as yet available, but the detection of these organisms may throw considerable light upon diagnosis. Recently, by this method, we found that a case which had been regarded as one of multiple tuberculous abscesses was in reality one of chronic pyæmia.

Staphylococci may also be found in the blood in acute periostitis. In typhoid fever it is generally not possible to demonstrate the Eberth-Gaffky bacillus in the blood, and the sample is collected for the purpose of performing Widal's test.

The bacilli of glanders, anthrax, and tetanus are best looked for in the discharges from the local lesion; for, although they may enter the general circulation, their detection in the blood is a matter of great difficulty.

**Method of Obtaining Specimens.**—Blood is best obtained either from the finger or the lobe of the ear. The skin is first washed with soap and water, defatted by rubbing with ether or turpentine, soaked with bichloride of mercury solution one in 1,000, and then cleansed with absolute alcohol. A ligature is tied round the root of the finger, which is then flexed to its full extent and punctured just above the root of the nail. The puncture is made with an ordinary fine sewing needle which has been sterilised by heating in the flame. The drop of blood thus obtained may be dealt with in different ways, according to the object of the examination. A portion should be received upon the sterilised platinum loop, to be spread out between coverglasses for immediate staining and microscopic examination, and a second portion inoculated on the surface of culture media.

If a larger quantity of blood be required, either as a sample for transmission to the laboratory or for the purpose of performing Widal's test, the method of procedure is as follows:—

A drop of blood having been allowed to accumulate over the puncture, one of the open ends of the capillary vaccine tube, which has previously been sterilised by heat, is inserted into it, and as much blood as can be obtained is allowed to run in. If a sufficient quantity is not got at the first attempt, the blood already collected may be driven further up the tube by shaking or the application of warmth, the finger squeezed, a second supply taken, and the ends of the tubes sealed by holding in the flame of a lamp or match. Some tubes provided for this purpose, especially those with a bulbous enlargement in the middle, require the blood to be sucked up into them, a procedure not altogether devoid of danger to the operator.

When preparing coverglass films of the malaria parasite the fixation should not be done by passing through the flame, as this would spoil the specimen. The blood having been spread out between two coverglasses, these are separated by sliding the one upon the other, and the films allowed to dry. They are then fixed by placing in a 5 per cent. solution of corrosive sublimate for five minutes, or in a mixture of equal parts of alcohol and ether for half an hour. They may then be stained with methylene blue for five minutes. To the unpractised eye the detection of the malaria parasite is often a difficult matter, and the safer course is to submit the blood specimens to expert examination.

Bacteriological methods, when applied to the detection of micro-organisms in the blood, must be conducted with most scrupulous attention to antiseptic precautions, for a very small degree of neglect may lead to grave error in diagnosis and treatment.

(*To be continued.*)

